# Optimising collagen scaffold architecture for enhanced periodontal ligament fibroblast migration

**DOI:** 10.1007/s10856-018-6175-9

**Published:** 2018-11-03

**Authors:** Jennifer C. Ashworth, Marco Mehr, Paul G. Buxton, Serena M. Best, Ruth E. Cameron

**Affiliations:** 1 Department of Materials Science and Metallurgy, University of Cambridge, 27 Charles Babbage Road, Cambridge, CB3 0FS UK; 2 Geistlich Pharma AG, Core Technologies, Bahnhofstrasse 40, CH-6110 Wolhusen, Switzerland; 3 Present Address: Stem Cell Glycobiology Group, Centre for Biomolecular Sciences, University of Nottingham, NG7 2RD Nottingham, UK

## Abstract

Design of cell-free scaffolds for endogenous cell recruitment requires an intimate knowledge of precise relationships between structure and biological function. Here, we use morphological analysis by Micro-CT to identify the key structural features necessary for periodontal ligament fibroblast recruitment into collagen scaffolds. By the combined use of time-lapse imaging and end-point invasion analysis, we distinguish the influences of pore size, pore wall alignment, and pore transport pathways (percolation diameter) on the individual cell migration and bulk invasion characteristics of these fibroblasts. Whereas maximising percolation diameter increased individual cell speed, elongation and directionality, and produced the most rapid bulk cell invasion, a pore size of 100 μm was found to be necessary to ensure an even distribution of cells across the scaffold cross-section. These results demonstrate that control of percolation diameter and pore size may be used respectively to tune the efficiency and uniformity of invasion through macroporous scaffolds. Crucially, however, these observations were subject to the condition of pore wall alignment, with low alignment in the direction of travel producing relatively low cell speeds and limited invasion in all cases. Pore wall alignment should therefore be carefully optimised in the design of scaffolds for cell recruitment, such as that required for periodontal ligament regeneration, as a key determining factor for cell movement.

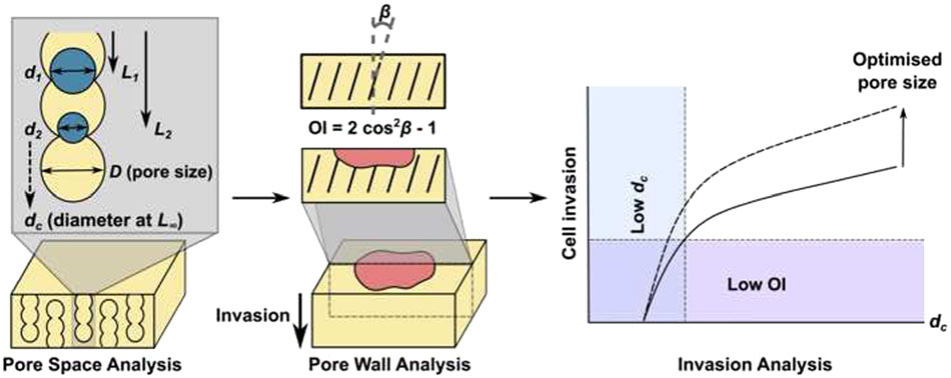

## Introduction

Understanding the structural cues presented to cells within a biomaterial scaffold has crucial implications for tissue engineering, as well as for the development of *in vitro* models of the extracellular matrix (ECM) [1–3]. Without an understanding of the vital link between material structure and cell behaviour, the design of novel biomaterials for specific applications will be based solely on intuition, or trial and error. Thorough characterisation of both biomaterial structure and cellular response is therefore paramount for ensuring the informed design of scaffolds for tissue engineering applications.

This is particularly important when applications with rigorous constraints on scaffold structure are considered. A key example is periodontal ligament (PDL) regeneration. The PDL fills the 200 μm gap between a tooth and its socket, providing support and vascularisation to the surrounding tissues [4]. Whereas progression of gum disease can lead to PDL destruction, and eventually to tooth loss [5], if PDL fibroblasts and their progenitors are able to re-enter the wound site, they can regenerate the original PDL space, complete with normal architecture of collagen fibres [6]. However, when designing a cell-free scaffold for recruitment of such cells, the dimensions of the PDL place an important constraint on the range of available pore sizes within any tissue engineering scaffold to be implanted into this space. It is therefore important to understand the necessary structural design criteria for cell invasion into these scaffolds.

There is a substantial body of research into the use of macroporous collagen scaffolds for tissue engineering applications, as compositional analogues of the ECM [7], [8]. These scaffolds are fabricated using a freeze-drying technique, which allows mimickry of ECM structure as well as composition, providing a biomimetic arrangement of structural and biochemical cues for cell attachment and migration [9–11]. Recent work has demonstrated that the structural characteristics of collagen scaffolds may be controlled to a much greater extent than previously acknowledged. In particular, it has been shown that pore size, anisotropy, and the availability of transport pathways are independently variable in collagen scaffolds, each with a distinct, cell-type specific influence on cell invasion [12–14]. The effects of such parameters on cell motility have been studied rigorously in isolation; for instance, it is known that lower pore sizes tend to inhibit cell dispersion towards the centre of scaffold constructs, whereas anisotropic scaffolds lead to elongated cells and enhanced migration relative to isotropic scaffolds [8, 15, 16]. However, a global understanding of the interplay between such parameters in determining cell behaviour is still evasive, as is the discernment of their relative effects. Without characterisation of every relevant structural feature, it is impossible to perceive which has the most influence in determining the observed cell response.

In this study, we show that collagen pore wall alignment in the direction of travel is a key requirement for periodontal ligament fibroblast (PDLf) migration, and that, subject to this condition, the speed and uniformity of PDLf invasion may also be tuned by careful control of pore structure. Using a set of collagen scaffolds with well-characterised variations in structure, we are able, for the first time, to test the relative influence of each feature of the pore space, and to correlate individual cell migration dynamics with overall cell infiltration. In addition to measurement of pore size, we use a technique recently developed in our lab to measure the object diameter able to traverse a scaffold of infinite size, the percolation diameter [12, 13]. This describes the transport characteristics in each direction through a scaffold, and therefore also provides a measure of scaffold anisotropy. Additionally, using bright field microscopy, we demonstrate that pore wall alignment may exist even in scaffolds with isotropic pore space. In this way, we are able to distinguish the separate influences of each parameter on PDLf motility, identifying possible strategies for optimised PDL regeneration by control of scaffold structural design.

## Materials and methods

### Scaffold fabrication

Collagen scaffolds were fabricated by freeze-drying suspensions of type I collagen from bovine Achilles tendon (Sigma-Aldrich, UK), as previously described [13]. Collagen was suspended at 1% (w/v) in either 0.05 M acetic acid (Alfa-Aesar, UK) or 0.001 M hydrochloric acid (HCl, Sigma-Aldrich), hydrated overnight and blended to a homogeneous mixture. This was poured into moulds made of stainless steel (both suspensions) or silicone (0.05 M acetic acid only), and freeze-dried using a computer-controlled protocol. Stainless steel moulds were cooled at 1.2 °C min^−1^ to −35 °C, silicone moulds were either cooled at 1.2 °C min^−1^ to −10 °C, or quenched to −20 °C. Mould filling was approximately 1 cm for the controlled cooling rate, or 2 cm for the quenched samples. After freezing was complete, the scaffolds were dried at 0 °C and 80 mTorr, before cross-linking using carbodiimide chemistry. Scaffolds were submerged in a solution of 1-ethyl-3-(3-dimethylaminopropyl) carbodiimide hydrochloride (EDC, Sigma-Aldrich) and N-hydroxysuccinimide (NHS, Sigma-Aldrich) in 95% ethanol, at a molar ratio of 5:2:1 (EDC:NHS:COOH), before drying using the same freeze-drying protocol as before.

### Morphological characterisation

The scaffold pore space was characterised in terms of pore size and percolation diameter, using Micro-CT imaging. A Skyscan 1072 system (Bruker, BE) was used to scan scaffold samples at 25 kV/137 µA, with a 7.5 s image acquisition time in rotation steps of 0.23°, averaged over four frames. The image pixel size was 3.74 µm. 3D datasets were obtained using the Skyscan software NRecon, and binarised using the Trainable Segmentation plugin in the ImageJ software distribution FIJI. Image noise was removed using the FIJI despeckle function in 2D, followed by a 2 × 2 × 2 median filter in 3D.

**Fig. 1:**
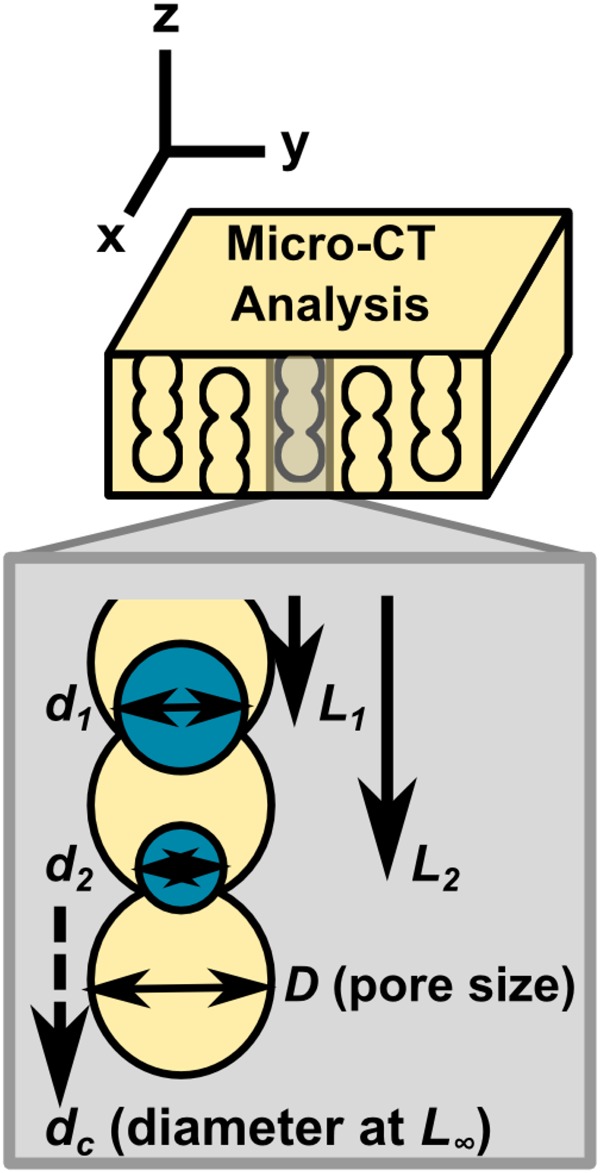
Schematic of characterisation methods, illustrating scaffold parameterisation in terms of *D* and *d*_*c*_, where *D* is mean pore size, and *d*_*c*_ is the percolation diameter, representing the characteristic size of the transport pathways in a given direction through the scaffold. Percolation diameter is calculated by successive measurements of *L* and *d*, where *L* is the accessible distance for an invading object of diameter *d*

Scaffold structure was parameterised by measurement of pore size, *D*, and percolation diameter, *d*_*c*_, as shown in the schematic in Fig. 1. Pore size was calculated from 2D slices of area 1 mm^2^, sampled at 50 μm spacing. Outliers up to 2 pixels in size were removed, and pore size was calculated as the diameter of the circle equivalent to each ellipse, fitted using the automated Watershed and Analyse Particles functions in the FIJI software. Percolation diameter, *d*_*c*_, which describes the characteristic size of the transport pathways through the pore structure in a particular direction, was also calculated for each scaffold, as previously described [12]. Measurements of accessible distance, *L*, as a function of invading object diameter, *d*, were made using the ROI shrink-wrap function in the Skyscan software CTAn, and fitted to the relationship in equation (1) in order to calculate *d*_*c*_:

1 $$L \propto (d - d_c)^{ - 0.88}$$

Average pore size and percolation diameter were measured from three different Micro-CT sub-volumes. Mean and standard error of these three measurements is reported.

### Real-time migration

Fluorescently labelled human periodontal ligament fibroblasts (PDLf) were obtained by stable transduction of primary PDLf (Lonza, CH) with CytoLight^TM^ Red Lentivirus (Essen Bioscience) and cultured in high glucose Dulbecco’s Modified Eagle Medium (DMEM, LifeTechnologies, CH) supplemented with 5% fetal bovine serum and 1% penicillin/streptomycin, with the addition of 0.5 µg/mL puromycin (P8833, Sigma-Aldrich). The cells were detached at sub-confluence with trypsin-EDTA. Scaffold samples cut to 10 × 10 × 2 mm^3^ were prepared for cell culture by sterilisation in ethanol, before washing in PBS and pre-wetting with medium. Seeding took place at passage 6, at a concentration of 20,000 cells in 20 µL medium per scaffold. One hour after seeding, scaffolds were transferred to a glass bottom 24-well plate, and extra medium was added. The scaffolds were then incubated at 37 °C and 5% CO_2_ in a humid atmosphere overnight, before transferring to the controlled atmosphere chamber in a Yokogawa CV1000 Cell Voyager confocal microscope system, which maintained the same conditions. Cell migration in the seeding plane was observed in real-time within each scaffold using time-lapse imaging, with images taken every 2 h over a 42 h period (day 1–3). Scaffold samples were turned over prior to imaging, such that the seeded surface was in contact with the base of the well plate.

The position of the fluorescently labelled cells was tracked manually over time, using the MTrackJ plugin in FIJI [17]. The plugin allows each cell position to be manually recorded at each time point, giving a measurement of the path followed by every cell. Ten such cell tracks were analysed for each scaffold condition, deliberately selected such that the cell remained visible within one focal plane for as many steps as possible. In this way, a measure of the migration characteristics of a cell moving within the seeding plane could be assessed. Cell tracks were visualised by plotting on a single plot for every scaffold, by overlaying the starting positions of each cell at the same origin. The speed of each cell was calculated by summing the distance travelled in each time interval, and dividing by the total time period for which the cell was visible. In this way, the mean and standard error of cell speed was calculated for each scaffold.

### End-point invasion

Untransduced PDLf were cultured using the methods described above (without puromycin addition), and seeded in triplicate for each time point onto the surfaces of pre-prepared scaffold samples, at a concentration of 15,000 cells in 1 µL medium per scaffold. The use of this small volume contained the cells within a small surface region in the centre of each scaffold. Culture conditions were maintained at 37 °C and 5% CO_2_ in a humid atmosphere with medium changed three times per week. Scaffolds were harvested at day 3 and 7 after seeding, by fixing with 10% formalin (Sigma-Aldrich) after an initial wash in PBS.

### Fluorescent staining and microscopy

Cells were permeabilised with a 10 minute incubation in 0.1% Triton X-100/PBS (Sigma-Aldrich), to allow cytoskeletal actin staining with Alexa Fluor® 488 Phalloidin (MolecularProbes, CH) at 2.5 µL/200 µL in 1% bovine serum albumin/PBS (BSA, Sigma-Aldrich). Additionally, the cell nuclei were stained using a 1:2000 dilution of DAPI in PBS (MolecularProbes, CH). Scaffolds were washed in PBS at each intermediate step. The stained scaffold surfaces were then imaged to reveal the position of the surface region containing the cells, to ensure that appropriate sections were taken from this approximate position. Scaffolds were sectioned by embedding in 15% gelatin/PBS (BioGel, CH), fixed with 10% formalin, and cut with a Leica VT1000 S Vibratome at 200 µm thickness to reveal the scaffold cross-section. Sections were imaged using the Cell Voyager microscope system described above, which recorded the maximum fluorescent intensity over 11 z-slices, spacing 20 µm, for each scaffold cross-section. Invasion distance at day 3 and day 7 was quantified by measurement of median cell position, as previously described [13]. Briefly, the fluorescent intensity profile was used to calculate the distance from the seeded surface at which the cumulative intensity was half its total value. The first 10 intensity values were not included in the calculation, to avoid the effect of high cell density at the scaffold surface producing unrepresentative values of median cell position within the scaffold itself. The mean value is given along with the standard error of six measurements.

The OrientationJ plugin in FIJI was used to quantify the collagen pore wall orientation from bright field images taken of the scaffold cross-sections. Using the method described by [18], this measurement was converted to an orientation index (OI), as defined in equation (2):

2 $$OI = 2cos^2\beta - 1$$

Where β is the angle between the direction of invasion (normal to the seeding plane) and the dominant direction of pore wall orientation, as illustrated in Fig. 1. According to this definition, OI is equal to -1 when pore wall orientation is perpendicular to the invasion direction (β = 90°) and equal to 1 when parallel to the invasion direction β = 0°). The mean value for each scaffold condition is given along with the standard error of six measurements.

### Statistics

Statistical significance was tested using one-way ANOVA, followed by a Tukey HSD test for pair-wise comparisons. Statistical significance was declared at *p* < 0.05.

## Results

### Scaffold structure

**Fig. 2:**
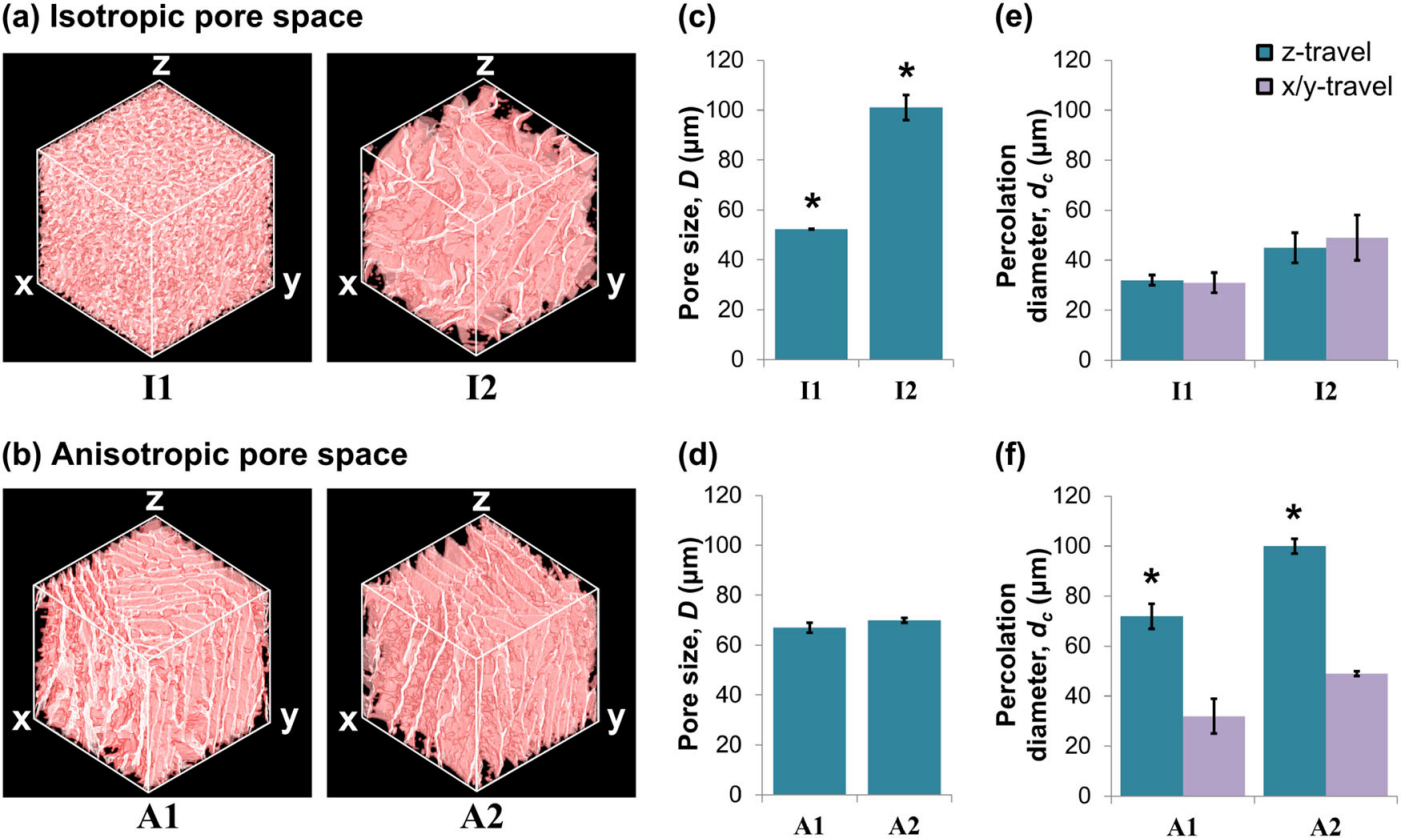
Micro-CT analysis of each scaffold structure. Representative 1 mm^3^ volumes (**a**, **b**) are shown in addition to the results of morphological analysis in terms of pore size, *D* (**c**, **d**) and percolation diameter, *d*_*c*_ (**e**, **f**) [13]. * indicates a statistically significant difference relative to all other conditions (*p* < 0.05)

Each of the four test scaffolds chosen for this study was imaged using Micro-CT to reveal their pore structure. Representative images are shown in Fig. 2a, b. Whilst two of the scaffolds (I1 and I2) had an isotropic pore space arrangement, the other two scaffolds (A1 and A2) contained highly anisotropic pore channels, giving pore wall alignment along the z-axis (the direction of freezing), as shown in Fig. 2. The characteristics of the pore space were parameterised as depicted in the schematic in Fig. 1, in terms of pore size, *D*, and also percolation diameter, *d*_*c*_, giving a measure of the transport properties in each direction through each scaffold. The results are shown in Fig. 2c–f, and in Table 1. Whereas neither scaffold I1 nor I2 showed direction dependence in their *d*_*c*_ measurements, scaffolds A1 and A2 both contained significantly higher values of *d*_*c*_ (*p* = 0.001) in the z-direction relative to the x/y direction (x and y are symmetrically equivalent in all scaffolds). Overall, the measured pore sizes ranged from 52–101 μm, while the *d*_*c*_ values spanned a range from 31-100 μm. Although these measurements correspond to scaffolds in the dry state, we have previously explored the influence of hydration on parameters measured by Micro-CT, and have determined that the effect is minimal in comparison with between-sample variation [19].

**Table 1:** Table summarising the ranges of pore size and *d*_*c*_ measured for each scaffold condition

Scaffold	Pore size (μm)	*d*_*c*_ for z-travel (μm)	*d*_*c*_ for x/y-travel (μm)
I1	52.3 ± 0.1	32 ± 2	31 ± 4
I2	101 ± 5	45 ± 6	49 ± 9
A1	67 ± 2	72 ± 5	32 ± 7
A2	70 ± 1	100 ± 3	49 ± 1

For a schematic of the methods used for measurement of each parameter, refer to Fig. 1. The standard error of the values calculated from three datasets is given, representing the reproducibility of each measurement method, rather than variability in structure

Although the pore sizes of scaffolds I1, A1 and A2 fell within a relatively small range, as reported previously [13], the difference in pore size between scaffold I1 (52 μm) and all other scaffolds was found to be statistically significant (*p* < 0.02). The pore size of scaffold I2 (101 μm) was also significantly larger than all other scaffolds (*p* < 0.001). Since scaffolds I1 and I2 had no significant difference in percolation diameter (*p* = 0.33), these scaffolds could therefore be used to examine the influence of an independent change in pore size on cell movement. Furthermore, since scaffolds A1 and A2 (*d*_*c*_ = 72 and 100 μm respectively) had a statistically significant difference in *d*_*c*_ but not pore size (*p* = 0.03 and 0.828 respectively), these scaffolds may be used to explore the independent influence of *d*_*c*_. In particular, *d*_*c*_ was greater than pore size in A2, which indicates the presence of a small number of interconnected channels with dimensions greater than the mean pore size. These four scaffolds may therefore isolate the separate influences of pore size and percolation diameter on cell behaviour, while also providing information on the effect of scaffold anisotropy.

### Single cell migration response to structure

**Fig. 3:**
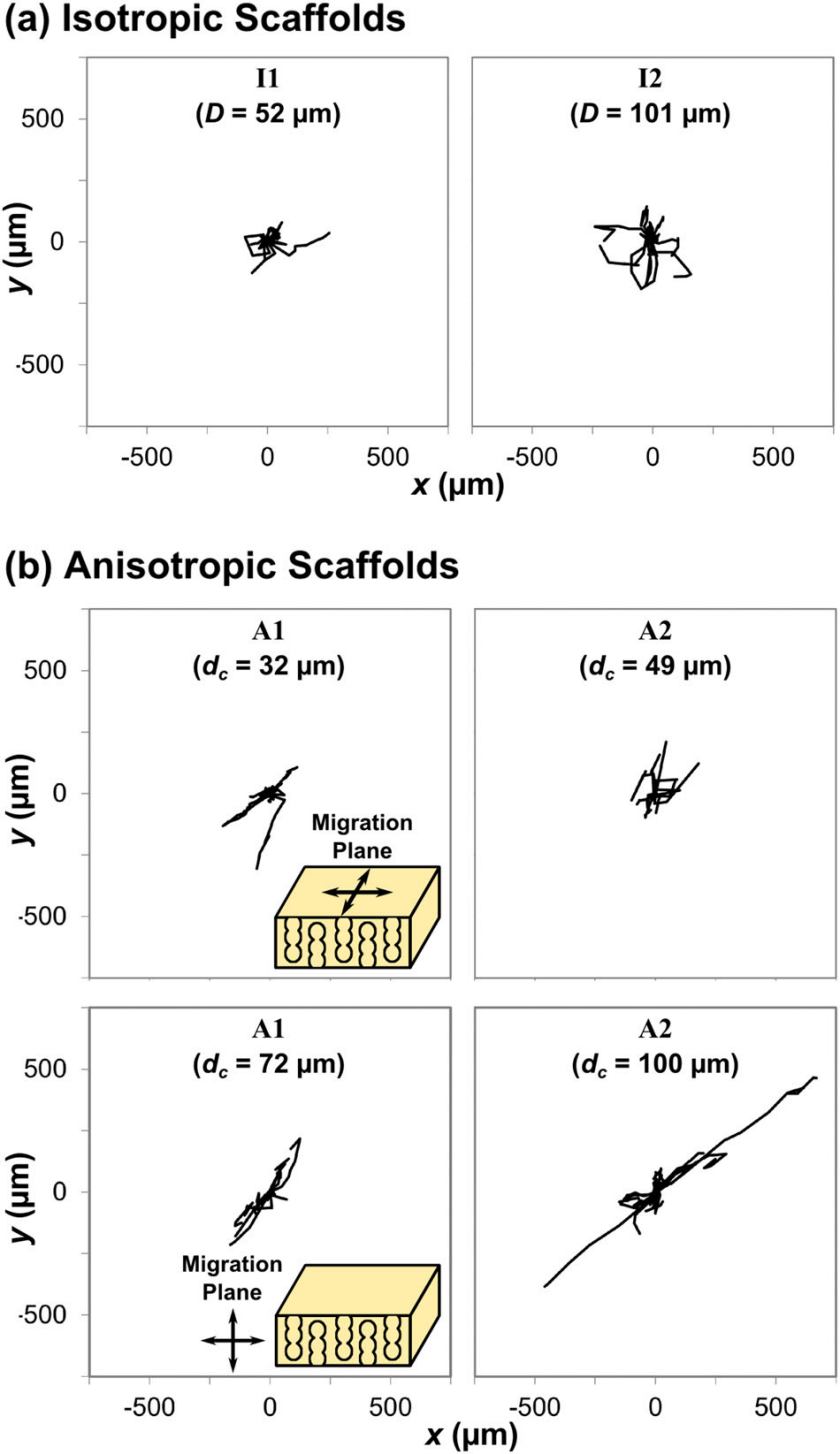
Migration tracks of 10 PDLf cells overlaid for **a** scaffolds I1 and I2, and **b** each plane of scaffolds A1 and A2, as shown in the schematics inset. The typical distance travelled by the cells remained relatively constant with pore size, *D*, but increased substantially where higher *d*_*c*_ was combined with pore wall alignment

To investigate the relative influences of pore size and *d*_*c*_ on the individual cell migration behaviour of PDLf, the positions of 10 cells were tracked over a period of 42 h within each scaffold (see Online Resources 1-6). The resulting wind-rose plots are shown in Fig. 3. As shown in the schematic, cell movement was tracked in two different planes for each of scaffolds A1 and A2, to account for their pore space anisotropy. Little difference was apparent between the cell tracks in scaffolds I1 and I2, shown in Fig. 3a, despite the considerable difference in pore size (52 and 101 μm respectively). Arguably, the cell tracks in I2 covered a larger area, but the maximum degree of displacement was very similar in each scaffold. However, the cell tracks in scaffolds A1 and A2, Fig. 3b, varied substantially according to both percolation diameter and imaging plane. Cells moving in the imaging plane containing the primary direction of pore wall alignment appeared to have greater directionality than those moving in the plane with no overall alignment. This is particularly evident in scaffold A2, *d*_*c*_ = 100 μm, in which the fibroblasts were able to travel much further distances relative to scaffold A1, *d*_*c*_ = 72 μm.

**Fig. 4:**
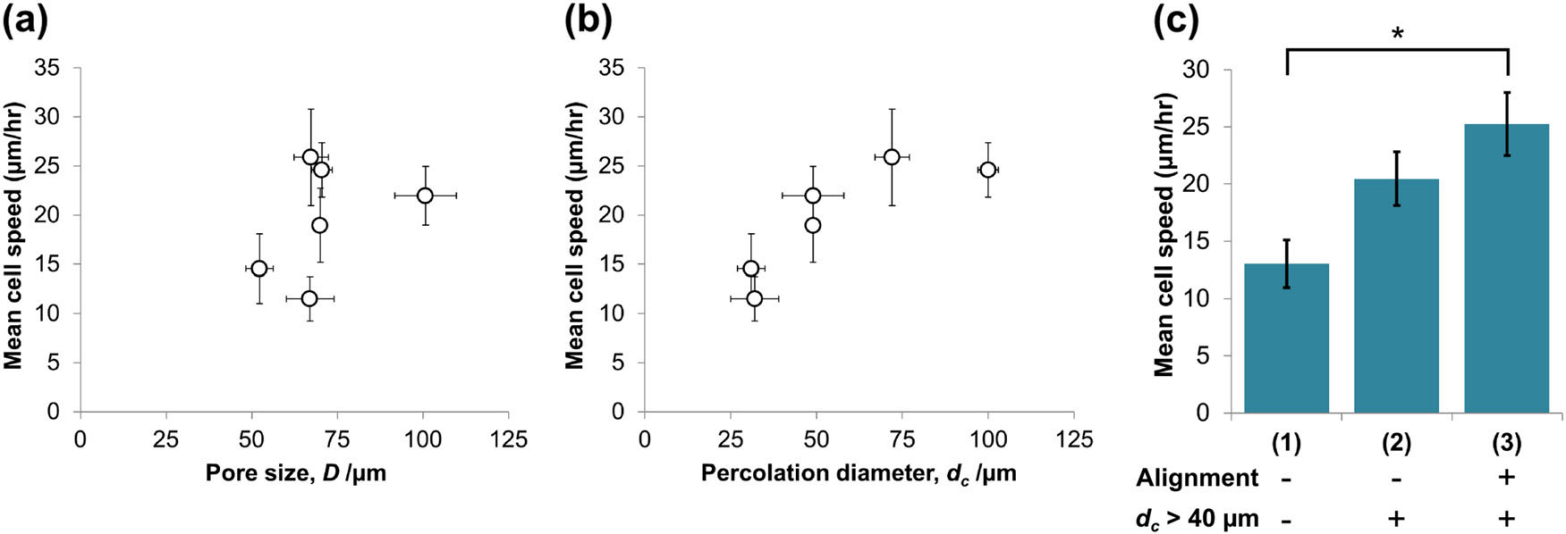
Mean PDLf migration speeds calculated for each of the cell tracks displayed in Fig. 3, plotted as a function of **a** pore size, *D*, **b** percolation diameter, *d*_*c*_, and **c** collated according to chosen structural criteria (presence of pore alignment and *d*_*c*_ *>* 40 μm in the direction of cell travel). * indicates statistical significance between two groups (*p* < 0.05)

For each cell track shown in Fig. 3, average speed was calculated by dividing the total distance travelled by the time period over which the cell was visible. The results are shown in Fig. 4, plotted as a function of both pore size and the maximum value of *d*_*c*_ in the imaging plane. While no correlation between cell speed and pore size was evident, an initial increase in cell speed followed by a plateau was observed when plotted against *d*_*c*_. This is striking in that it resembles a previously reported finding: that a *d*_*c*_ greater than 40 μm is required for fibroblast invasion in scaffolds of constant pore wall alignment [13]. To account for the complicating factor of variation in pore wall alignment between the scaffold conditions in the present study, the cell tracks from Fig. 3 were subdivided into three groups, according to the local structural properties: (1) *d*_*c*_ < 40 μm, (2) *d*_*c*_ > 40 μm with no pore wall alignment in the imaging plane, and (3) *d*_*c*_ *>* 40 μm with aligned pore walls (verified visually in bright field). The comparison between the cell speeds in these three groups is shown in Fig. 4c. The average cell speed steadily increased from groups (1) to (3), with the difference between groups (1) and (3) found to be statistically significant (*p* = 0.002). This provides evidence that the single cell migration speed of PDLf may be increased by optimisation of both pore wall alignment and *d*_*c*_.

### Bulk cell invasion response to structure

**Fig. 5:**
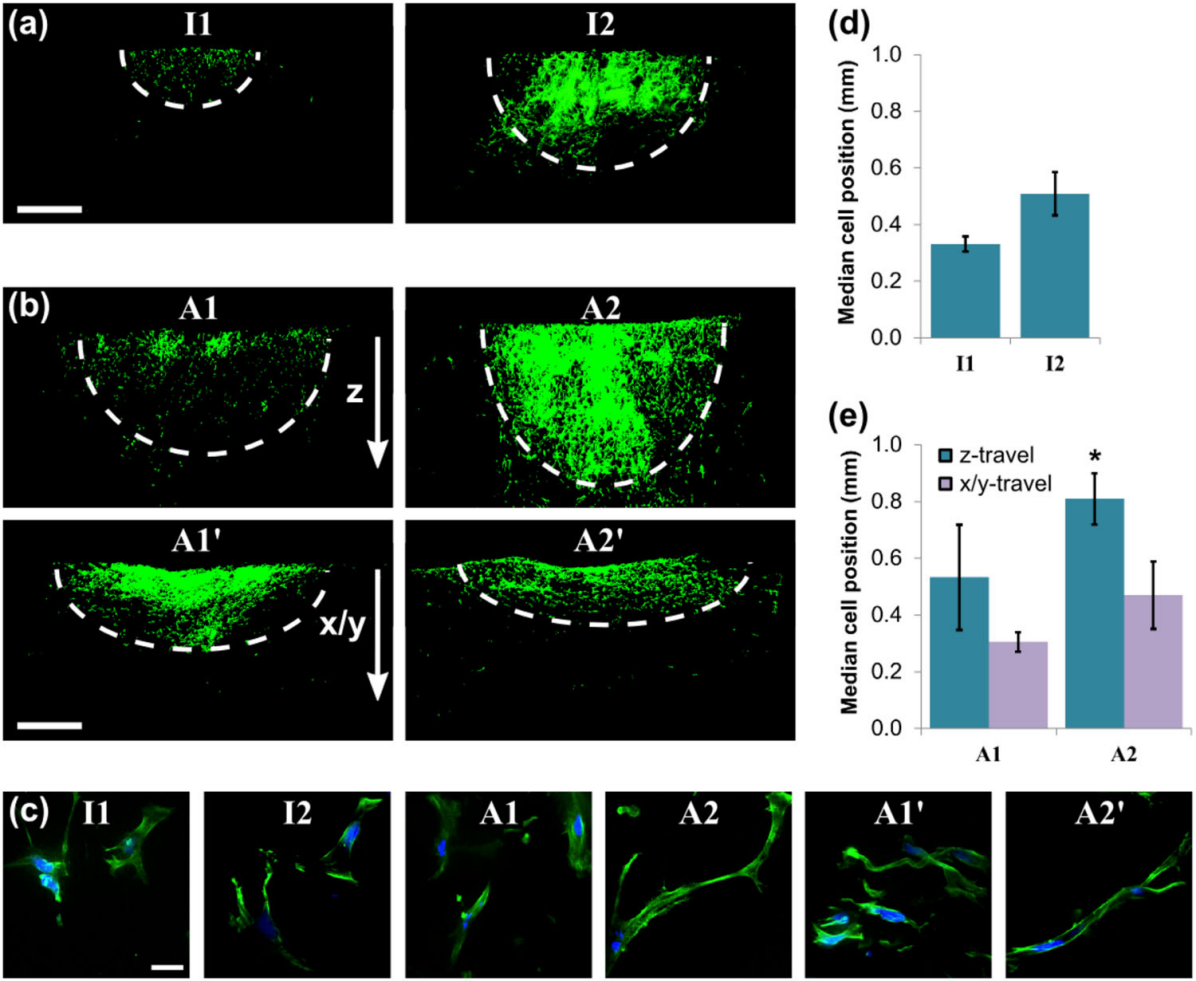
PDLf invasion at day 3 after seeding, revealed by Phalloidin F-actin staining for **a** the isotropic scaffolds I1 and I2, and **b** the anisotropic scaffolds A1 and A2, scale bar 1 mm. Individual cell morphology is also shown (**c**, scale bar 25 µm), along with quantification of invasion in terms of median cell position (**c**, **d**). * indicates statistical significance relative to the two lowest measured values

End-point analysis of cell position allowed the influence of scaffold structure to be examined on the scale of the overall cell population. The chosen method of seeding a small volume of cell suspension on the scaffold surface resulted in a roughly semi-circular shape to the dispersed spot of cells when shown in cross-section. Figure 5a and b show the shapes of these spots at day 3 after seeding. The seeding surface of the anisotropic scaffolds A1 and A2 was defined such that the aligned pore walls were directed into the scaffold, perpendicular to the seeding surface. However, to examine the effect of anisotropy, additional samples from these scaffolds were oriented such that the pore walls were aligned parallel to the surface. Scaffolds seeded in this way were labelled A1’ and A2’, as shown in Fig. 5b. This seeding method caused a change in the symmetry of the spot of cells, which extended along the scaffold surface with very limited invasion into the scaffold depth. As shown in Fig. 5c, the shape of the cell spot was also closely related to the morphology of the individual cells, which were generally elongated along the long axis of each spot. As well as varying with scaffold anisotropy, the depth that the cells could penetrate into the scaffold interior also increased with both pore size and percolation diameter. In particular, the size of the spot of cells increased between scaffolds I1 and I2, as shown in Fig. 5a, and also between scaffolds A1 and A2, as shown in Fig. 5b. In scaffold A2, which was characterised by anisotropic pore space, aligned pore walls and the largest percolation diameter tested (*d*_*c*_ = 100 μm), some cells were able to infiltrate into the full depth of the scaffold. Notably, Fig. 5c shows that the cells within this scaffold were far more elongated than in the other conditions. However, as shown in the bar charts in Fig. 5d, e, the median cell position (distance from the seeding surface) was measured to be less than 1 mm in all scaffolds. This indicates that at this early time point, the majority of the cells are close to the seeding surface in all scaffolds.

**Fig. 6:**
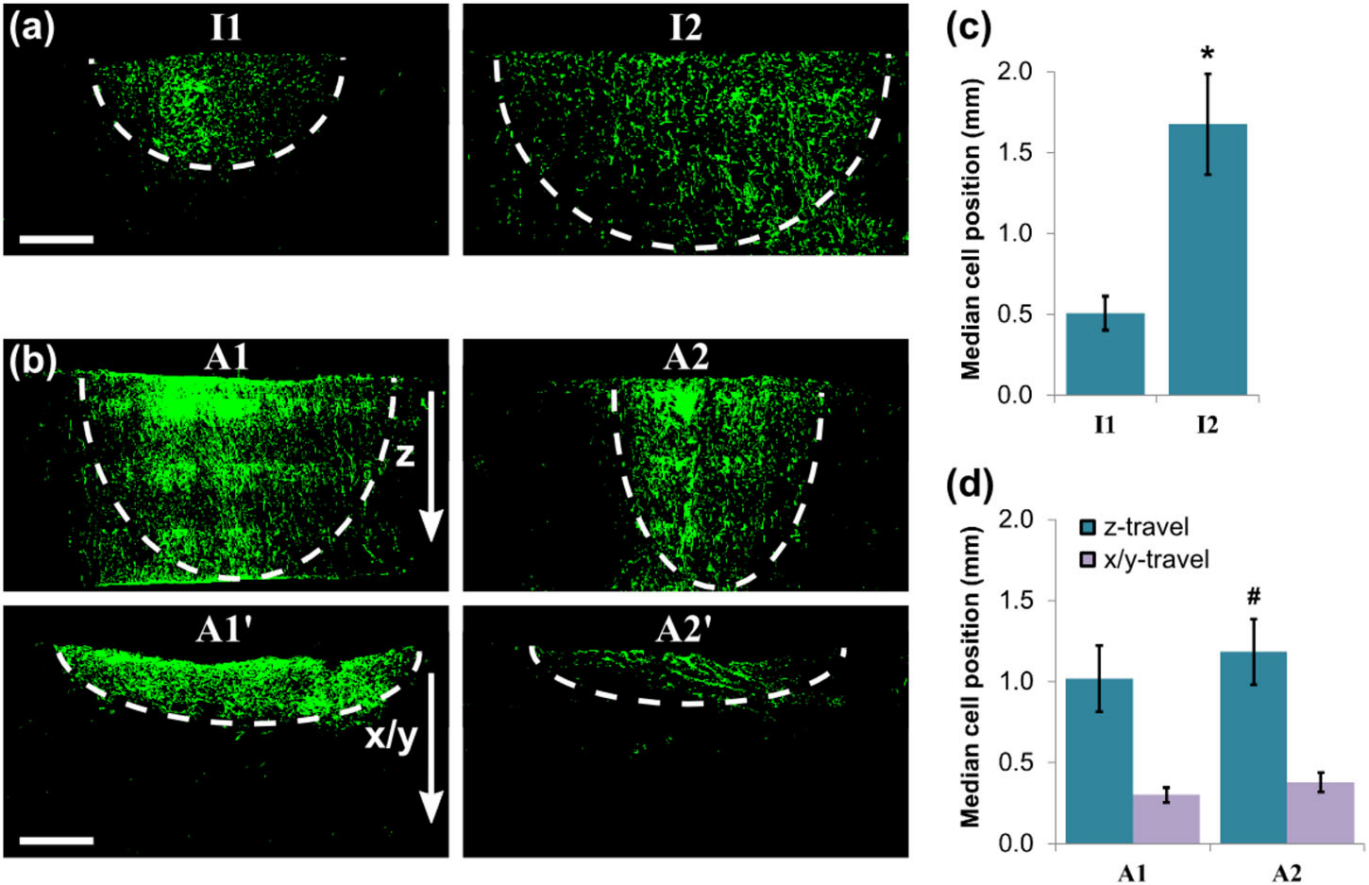
PDLf invasion at day 7 after seeding, revealed by Phalloidin F-actin staining for **a** the isotropic scaffolds I1 and I2, and **b** the anisotropic scaffolds A1 and A2, also shown quantified in terms of median cell position (**c**, **d**). Scale bar 1 mm. Note that the bar chart y-scale in (**c**) and (**d**) has doubled relative to Fig. 5d, e. # and * indicate statistical significance relative to the lowest two and three measured values respectively

At day 7, however, the influence of scaffold structure on PDLf invasion could be more easily differentiated. Figure 6a, b show representative images of the dispersed spot of cells, shown in cross-section for each scaffold. It is clear that a fraction of the cell population was able to invade through the entire scaffold thickness in all of scaffolds I2, A1 and A2. In scaffold I1, however, the degree of cell invasion was far lower. Additionally, Fig. 6b shows that when the anisotropic scaffolds were oriented with their pore walls along the scaffold surface (scaffolds A1’ and A2’), cells were unable to invade further than 1 mm from the surface. Similar patterns are seen when median cell position relative to the seeding surface is considered, as shown in Fig. 6c, d. This provides a measure of the proportion of cells that were able to invade efficiently through each scaffold. The greatest measured values of median cell position were seen in scaffold I2, the scaffold with greatest pore size (*D* = 100 μm). It therefore appears that both pore size and the arrangement of collagen pore walls play a role in determining the invasion response of the overall cell population.

**Fig. 7:**
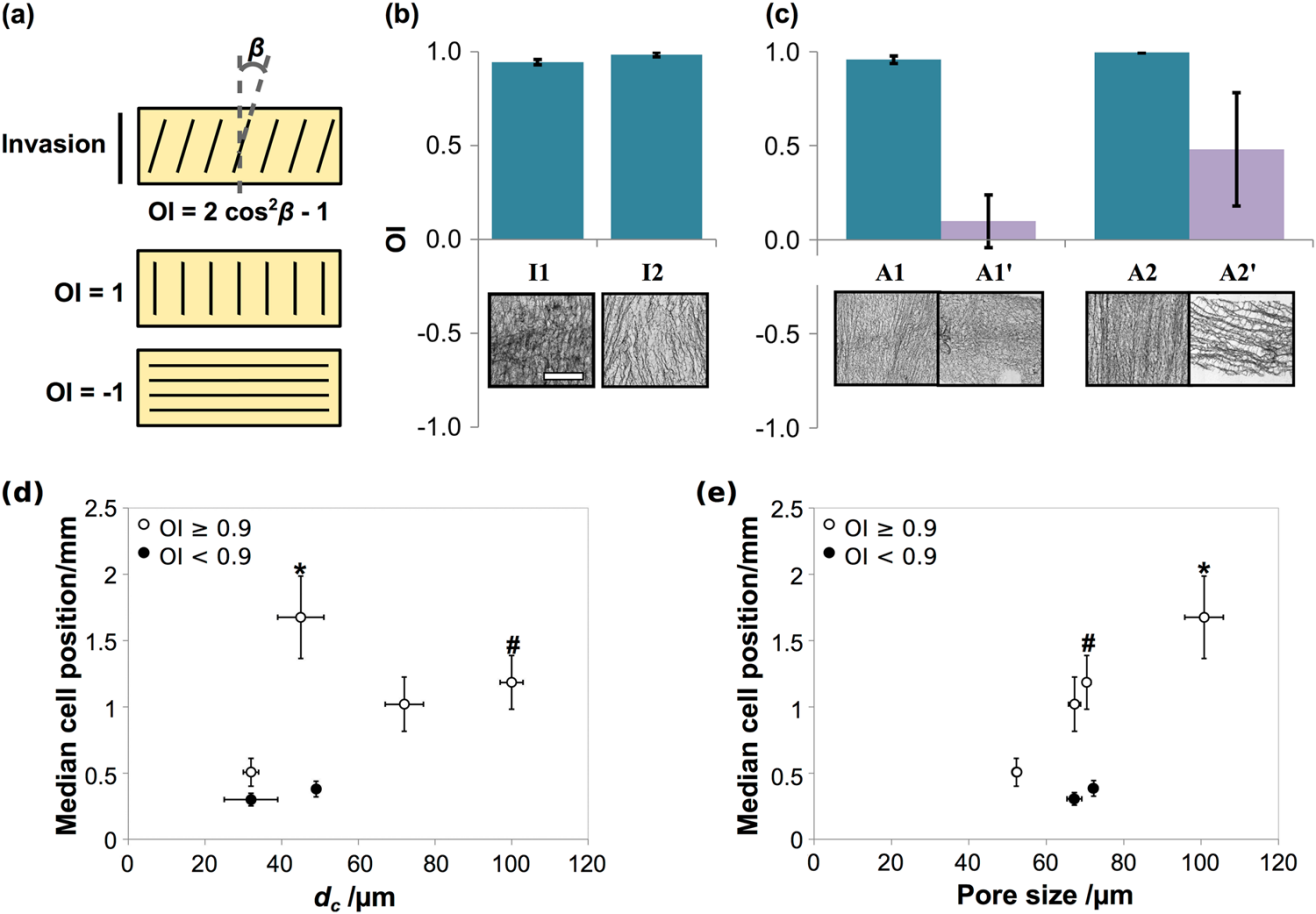
Pore wall orientation analysis from bright field microscopy, quantified in terms of orientation index, OI as depicted in the schematic in **a**. Measurements of OI **b**, **c** revealed that all scaffolds (except A1’ and A2’) contained pore wall orientation in the direction of invasion (OI > 0.9). Plotting the invasion results from Fig. 6 as continuous data series with constant OI, as in figures **d** and **e**, reveals that at high OI, median cell position at day 7 was primarily determined by pore size. # and * indicate statistical significance relative to the lowest two and three measured values (*p* = 0.04 and *p* = 0.001) respectively. Scale bar 1 mm

To examine this relationship more closely, each scaffold cross-section was imaged in bright field, revealing the local orientation of the scaffold pore walls. Orientation index, OI, was also calculated for each image, as shown in the schematic in Fig. 7a. Scaffolds A1’ and A2’ contained high variability in their OI measurements, according to the local pore wall orientation in the sectioning plane, which did not necessarily contain the primary direction of pore wall alignment. Importantly, however, as shown in Fig. 7b, c, some degree of pore wall anisotropy was apparent in all scaffolds, even I1 and I2, which appeared isotropic when imaged using Micro-CT. Quantitative analysis revealed that the global pore wall orientation was, in fact, constant in all of scaffolds I1, I2, A1 and A2, with an orientation index of OI = 0.94 or above (OI = 1 indicates perfect vertical alignment). Therefore, any differences in cell invasion behaviour in these scaffolds must be as a result of differences in pore space properties.

Figure 7d, e display the median cell position data from Fig. 6, as plots against percolation diameter and pore size respectively, separated according to the pore wall orientation, measured as OI. It is clear from these plots that, subject to the condition of pore wall alignment in the direction of invasion, median cell position followed a general linear trend with increasing pore size up to 100 μm. Taken together, these results indicate that although a larger percolation diameter may ensure fast and efficient PDLf infiltration through a scaffold, a pore size of 100 μm ensures the most even distribution through the cross-section. Most crucially, however, these relationships only hold true when the collagen pore walls are directed into the depth of the scaffold.

## Discussion

Since the restricted dimensions of the periodontal ligament place practical constraints on the pore size of a scaffold used to fill this space, it is important to understand the link between structure and cell movement beyond the effects of pore size. The presence of aligned collagen structures has often been observed to improve cell motility, with suggested mechanisms including mechanical anisotropy, contact guidance and molecular-scale topography [20–22]. In previous work, we have also demonstrated the importance of interconnected pore transport pathways for ensuring directed cell movement [13]. By combined characterisation of these distinct parameters, we have shown that efficient PDLf invasion may be achieved even in scaffolds of relatively low pore size (below 100 µm), provided percolation diameter and pore wall alignment are optimised.

In the presence of pore wall alignment, increasing percolation diameter was associated with increased cell displacement (Fig. 3), speed (Fig. 4) and elongation (Fig. 5). Directed cellular alignment according to the arrangement of structural features has been previously observed in a range of collagen structures, from dense gels [23] to highly porous scaffolds such as those investigated here [24]. An elongate cell morphology is known to be important for directed cell migration, as well as being highly sensitive to structure [25]. It therefore appears that a structure with high pore channel alignment and high *d*_*c*_ provides sufficient structural cues to allow efficient cell elongation and therefore continuous, persistent cell motion. This may result from a decrease in erratic cell movement, which is known to occur in the presence of competing structural cues for migration, for instance in random systems or at pore strut junctions [20, 26].

Whereas cell infiltration may intuitively be enhanced by maximising pore size, previous studies have shown that the determining factors for individual cell movement may be more complex. In particular, Harley *et al*. have previously shown that a decrease in pore size from 151 to 96 μm increases individual cell speed and dispersion, by increasing the density of ligands available to migrating cells [26]. Our results indicate that decreasing the pore size from 100 μm down to a minimum of 52 μm does not produce any further change in cell migration dynamics. This could indicate that a pore size close to 100 μm is optimum in terms of maximising cell speed and displacement. More crucially, however, the greatest cell speed and directionality in this study were observed in response to aligned pore walls, rather than to changes in pore size.

On examining cell movement at the scale of the bulk population, we observed that a structure with high anisotropy, pore wall alignment and high *d*_*c*_ permitted extensive PDLf invasion by day 3. This indicates that increased speed and directionality on the scale of the single cell could predict the efficiency of overall cell invasion into a scaffold. A further correlation between individual cell migration and bulk cell invasion may also be found by examining Fig. 4b. The relationship between cell speed and percolation diameter, an initial increase followed by a plateau, is very similar to the relationship between bulk cell invasion and percolation diameter described previously, with a plateau at *d*_*c*_ = 40 μm [13]. Step changes in cell migration dynamics have previously been observed when the length scale of obstructions approaches the cell size [27]. It is therefore likely that low percolation diameters correspond to structures in which the density of obstacles is too great to allow unhindered cell migration. Since cell migration at pore strut junctions has previously been observed to be slower than along pore struts [26], it is probable that the drop in cell speed at low *d*_*c*_ corresponds to less time spent moving along open pore struts, which are likely to be less available in occlusive structures.

By day 7, all of scaffolds I2, A1 and A2 permitted PDLf invasion through the entire cross-section. This demonstrates that pore sizes greater than 100 µm are not necessary for ensuring complete cell invasion. However, the highest median cell position corresponded to the scaffold with highest pore size, I2, which indicates that high pore sizes may be beneficial for improving the uniformity of the cell distribution. Where the pore walls and channels were directed along the scaffold surface (scaffolds A1’ and A2’), very limited invasion was observed into the bulk of the scaffold. Furthermore, no increase in cell invasion was seen between days 3 and 7. Where pore wall alignment was directed into the scaffold, however, even the low pore size, low *d*_*c*_ structure of scaffold I1 produced higher invasion at day 7 compared with day 3. Therefore, although control of pore structure can optimise the extent and rate of PDLf invasion, the presence of pore wall alignment appears to be the key limiting factor.

Bright field microscopy also revealed that the collagen pore walls were highly oriented in the invasion direction in each of scaffolds I1, I2, A1 and A2. This is likely to be caused by the nature of the freeze-drying technique used to fabricate the scaffolds: directionality in the freezing step could result in alignment of the resulting collagen pore walls [28]. This is supported by the fact that no pore wall alignment was observed in scaffolds I1 and I2 in the real-time migration assay, since here the scaffolds were seeded in the plane perpendicular to the freezing direction. However, this highlights the need for combined analysis of pore structure (by Micro-CT) and pore wall structure (by bright field microscopy) to fully characterise scaffold anisotropy. Here, this approach has revealed that the combined presence of aligned pore walls and a pore size of 100 μm produced the most uniform cell dispersion by day 7.

**Fig. 8:**
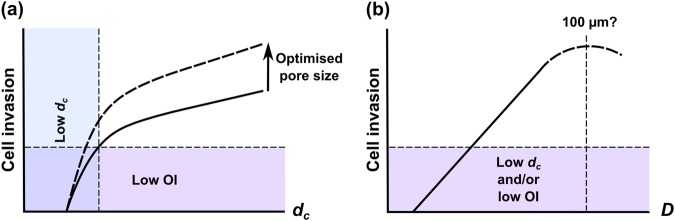
Illustration of the key relationships between scaffold structure and PdLf invasion observed in this study, shown as a plot against **a** percolation diameter, *d*_*c*_, and **b** pore size, *D*. So long as pore wall orientation (OI) and *d*_*c*_ are optimised, the extent of cell invasion follows a trend with *D*, up to the maximum of 100 μm tested in this study

Taken together, the results of this study show that, when pore wall alignment is directed into the bulk of a scaffold, cell invasion becomes faster and more efficient on maximisation of *d*_*c*_, and more uniform as pore size is increased up to 100 μm. The illustration in Fig. 8 draws these concepts together, providing a possible interpretation of the various structural influences on cell invasion. The first key concept is that, regardless of pore size or *d*_*c*_, substantial PDLf invasion can only occur when the pore walls are aligned in the direction of invasion. Therefore, the minimum structural requirement for PDLf invasion is pore wall alignment in the desired invasion direction. If this requirement is fulfilled, the speed and extent of invasion then becomes dependent on pore structure. The efficiency of cell movement is enhanced by maximising *d*_*c*_, ensuring it is sufficient to allow unimpeded invasion through the whole scaffold cross-section. In particular, we have previously reported that the condition *d*_*c*_ = 40 µm must be exceeded to ensure efficient fibroblast invasion, which is in agreement with the cell speed data in Fig. 4 [13]. As long as these two criteria are met, the ability of the cells to distribute themselves evenly throughout the scaffold cross-section scales primarily with increasing pore size, at least within the structural ranges tested in this study. As discussed above, it may be that an optimum pore size exists at a value of around 100 µm, since this was seen to produce the maximum level of invasion in this study, and has also been associated with high individual cell speed and dispersion in previous work [26].

The speed at which resident cells are able to colonise a tissue engineering scaffold has crucial importance for the efficacy of regeneration, since their presence is a necessity for tissue formation [29]. Although longer-term studies into scaffold-induced differentiation and matrix production would also be required to identify an ideal structure for PDL regeneration, reliable cell infiltration is the first step towards ensuring the long-term regenerative potential of these scaffolds. The requirement for cell invasion has previously been shown to influence the long-term osteogenic potential of collagen scaffolds, with human osteoblasts requiring at least 21 days to become homogeneously distributed in scaffolds of pore size 96 μm, with matrix deposition then initiating at the scaffold edges [30]. Here we have demonstrated the dual necessity of collagen pore wall alignment and high percolation diameter in the invasion direction for ensuring fast, directed PDLf migration, and therefore efficient invasion within 7 days. Although pore size also plays a role in ensuring a uniform cell distribution, here we have shown that PDLf are able to invade into the full thickness of tissue engineering constructs with a pore size of less than 70 µm, when pore wall orientation and percolation diameter are optimised. This is a key result for the design of scaffolds for PDLf recruitment, in that it provides a shortlist of scaffold structures for *in vivo* optimisation. The *in vitro* results presented here indicate that any of scaffolds I2, A1 and A2 would be suitable starting points for the design of a scaffold for PDL regeneration, so long as the pore wall orientation was carefully aligned with the desired direction of cell movement. This provides the flexibility to consider other physical constraints on scaffold structural design, such as tissue dimensions in the case of the PDL, as well as providing sufficient surface area for attachment and space provision for tissue synthesis. Optimising these parameters is likely to accelerate the entire process of tissue regeneration, by allowing fast and efficient endogenous cell recruitment.

## Conclusions

Although collagen scaffold structure is known to influence cell motility, to date, the relative importance of each structural feature has not been explored. Using rigorous scaffold characterisation techniques, we have identified the key structural features underpinning both individual cell migration dynamics and bulk invasion of periodontal ligament fibroblasts. At the scale of the individual cell, combined optimisation of pore wall alignment and percolation diameter was found to increase cell speed, directionality and elongation. At the scale of the bulk cell population, this effect also extended to faster invasion. Furthermore, the uniformity of the cell distribution was enhanced by increasing the pore size from ~50 to 100 μm, but only when the above criteria were also met. In particular, pore wall alignment in the desired direction of invasion was found to be a crucial limiting factor, with other structure-function relationships suppressed in its absence. This is a necessary, but not sufficient criterion for PDLf invasion, with optimisation of the pore space required for maximum cell infiltration. These results provide an enhanced understanding of the optimum biological environment for periodontal ligament regeneration, allowing fine-tuning of cell recruitment into tissue engineering scaffolds by control of their structure.

## Electronic supplementary material

Online Resource 1

Online Resource 2

Online Resource 3

Online Resource 4

Online Resource 5

Online Resource 6

Online Resources
