# Supplementary material for: Optimising collagen scaffold architecture for enhanced periodontal ligament fibroblast migration
Source: J Mater Sci Mater Med. 2018 Nov 3;29(11):166. doi: 10.1007/s10856-018-6175-9 (PMC6223802; doi:10.1007/s10856-018-6175-9)
Supplement: Supplementary file 7 — Online Resources [file 10856_2018_6175_MOESM7_ESM.docx]

**Online Resources**

Time-lapse microscopy images taken every 2 hours over a 42 hour period are provided as raw data in .avi movie format. Fluorescently labelled cells are shown in red, overlaid onto the bright field images of the scaffold structure. The files provided are as follows:

- Online Resource 1: Scaffold I1: cells moving in-plane within isotropic scaffold I1 (pore size 52 µm)
- Online Resource 2: Scaffold I2: cells moving in-plane within isotropic scaffold I2 (pore size 101 µm)
- Online Resource 3: Scaffold A1 (anisotropic): cells moving within scaffold A1 in an anisotropic plane (*d_c_* = 72 µm)
- Online Resource 4: A1 (isotropic): cells moving within scaffold A1 in an isotropic plane (*d_c_* = 32 µm)
- Online Resource 5: A2 (anisotropic): cells moving within scaffold A1 in an anisotropic plane (*d_c_* = 100 µm)
- Online Resource 6: A2 (isotropic): cells moving within scaffold A2 in an isotropic plane (*d_c_* = 49 µm)

*d_c_* refers to the percolation diameter in the direction of travel, as defined in the main text.

NB: Movies represent raw data, and as such the sample name annotations on the images themselves do not match the scaffold names used in this manuscript – refer instead to the list above.
